# Biomedical journal editing: elements of success

**DOI:** 10.3325/cmj.2011.52.423

**Published:** 2011-06

**Authors:** Armen Yuri Gasparyan, Lilit Ayvazyan, George D. Kitas

The current pace of developments in virtually every aspect of our life and scientific innovations pose ever increasing challenges in ensuring the highest possible quality of publications, satisfying the needs of both publishers and readers. Scholarly journals are essential tools for communication between experts and for advancement of research and practice in various fields of science (1). By communicating original research data, comprehensively covering emerging scientific concepts and directions and analyzing news reports, journals are being increasingly recognized as educational tools. Relevant examples are top general medical journals, such as *The Lancet, The New England Journal of Medicine,* and *The British Medical Journal*, reflecting developments in science and educating physicians and eventually changing clinical practice worldwide. Multiple functions assigned to scholarly journals raise the issues of trustworthiness and quality of the publications. The latter is of particular importance in view of recent trends in information flow, digitalization, and acceleration of the publishing process, which may increase the rate of errors and mistakes.

## Journal funding

The quality of a journal is subject to a variety of financial, infrastructural, technical, and scientific factors. Undoubtedly, secure financial sources for editing and publishing is *sanctum sanctorum* for any journal. Journals supported by learned societies, academic institutions, and leading publishers are in a privileged position, as they can afford to support the maintenance of editorial offices and secretarial services, prerequisites of a successful journal (2). The list of activities requiring financial support and incentives is long, and therefore priority should be given to activities yielding the greatest outcome for the investment (eg, digitization and indexing of a journal archives, awarding the most active contributors, etc.).

## Editorial team

The editorial office should be supported by a team of devoted and qualified editors and consultants. Remarkably, prestige and opportunities for indexing of a journal are subjected to the list of experienced and pro-active editors, who demonstrate skills in improving each and every section of the published items (3). The shorter list of editors and consultants, the better and quicker editorial work can be achieved, particularly in small journals.

The tasks of scientific and technical editors should be strictly defined, with expected regular contributions from each member of the editorial team. The most desirable and useful contribution of scientific editors and distinguished members of the editorial board is the submission of publishable manuscripts. The latter is especially important for new and small journals, where great publications by eminent scientists and authors can boost the journals’ profile and attract many new submissions of similar quality. Even at some well-established journals, membership of the Editorial Board implies an obligation to regularly (at least once a year) submit good manuscripts plus invited editorial commentaries. No less important are the editors’ efforts to improve editorial policy, the quality of peer review, and the validity of publications. Editorial board meetings should be organized regularly to discuss such matters. Most top general medical journals such as *The Lancet* and *BMJ* organize weekly meetings to present submissions, outcomes of the peer-review, and many other issues. For smaller and specialized journals that publish fewer issues per year, weekly meetings are not necessary; annual or biannual discussions on issues related to editorial policy, quality, peer-review, and indexing may be more appropriate.

The criteria for editors’ qualifications vary between general and specialized journals and from country to country, but, undoubtedly, most journals would benefit from recruiting experts familiar with international standards of science writing and editing, members of learned societies such as the Council of Science Editors (CSE), the World Association of Medical Editors (WAME), the European Association of Science Editors (EASE), and those accredited as professional editors (eg, those who passed the Board of Editors in the Life Sciences [BELS] exam). In addition, it is becoming increasingly important to adhere to the principles of ethical publishing outlined in the guidelines of the Committee on Publication Ethics (COPE), and most journals and individuals involved in science editing are encouraged to join COPE. Many leading publishers pay a group subscription to COPE for all their journals, eg, Elsevier, Wiley-Blackwell, and Informa Healthcare.

For English language journals, particularly those published in non-Anglophone countries, language editors with qualifications from leading linguistic institutions in Oxford, Cambridge and elsewhere, membership in relevant associations (ie, American Medical Writers Association [AMWA], European Medical Writers Associations [EMWA]), and skills in science editing are valuable assets. Language editing, correcting grammar and style of the titles, abstracts and key words can be the first step toward enhancing the quality of publications and improving the influence of the papers. Some high-impact journals even employ title and abstract editors (4). Editors of new, small and non-English journals struggling with indexing in general online databases such as Web of Science are well aware that correctly structured, informative, and reflective titles and abstracts are the ‘magic’ keys to successful journal indexing (5).

## Internationalization of peer review

Depending on the rate of manuscript submissions and their quality, journal editors may choose to rely on either internal or external peer-review or both. Internal processing of the submissions is primarily aimed at filtering out manuscripts with irrelevant scope, incomprehensible language and narrative, and apparent errors in scientific design and methodology. Rapid internal review is a good service to the authors and, in case of rejections, is a tool for saving editors’ and external reviewers’ time and efforts (6). For journals struggling with quality and indexing issues, particularly for those published by national professional societies in non-Anglophone countries, internationalization of the peer-review process and involvement of skilled reviewers in the evaluation of scientific, linguistic, and technical aspects of submissions is a way toward high publishing standards and wider visibility (7-9). Importantly, one of the basic requirements for inclusion in prestigious databases such as MedLine and Science Citation Index or Social Science Citation Index is high-quality, unbiased, and comprehensive peer-review, which can be best organized by inviting experts with relevant professional background and active in writing different types of scientific articles and reviewing for international journals (10). Good reviewer comments should be ethically sound, constructive, sufficiently detailed, comprehensive, educational, and confidential during the whole process of the peer-review (11). Evidence suggests that most reviewer comments of high quality, particularly in the biomedical field, are those written by young postgraduates with up to 10 years in practice and academics from university affiliated hospitals (12). Obviously, there is a huge shortage of skilled and available reviewers, and publishers and editors should permanently strive to attract the best possible reviewers (13). Once a reviewers’ database has been established, the least editors can do to reward efforts and to stimulate regular and new reviewers’ interest toward writing great comments is to publish annual acknowledgments, perhaps distinguishing the most productive contributors. Publicizing the list of reviewers, information on timelines of the peer-review, and rates of rejected and accepted manuscripts can be also viewed as indicators of transparency and journal quality (5). Relevant examples are the open access electronic journals published by DovePress, where each journal’s Web site displays article processing statistics and a regular list of reviewers.

## Scope, coverage, and content

Highly selective databases such as the Science Citation Index and MedLine/PubMed place a great importance on the originality of the journals applying for indexing. This implies a unique journal title, specific scope of interest, original content of the published articles, and defined professional and geographical representation. Additional value is given by content of international importance and of interest to scientists around the world, even if publications are based on national/local studies.

Correctly chosen journal titles reflecting a specific subject category, and geographical and societal affiliation are critical for attracting new submissions relevant to the declared scope of interests. Good examples of general journals covering a variety of scientific fields are *Proceedings of the National Academy of Sciences of the United States of America*, *Science,* and *Nature*. Exemplary are titles of the journals belonging to professional societies in certain regions: *Journal of the American College of Cardiology, Journal of the Royal College of Physicians of Edinburgh,* etc. Editors expecting the majority of submissions from their academic and/or clinical institutions rightly choose titles indicating journal affiliation (eg, *Texas Heart Institute Journal, Journal of Tehran University Heart Center, Journal of Zhejiang University SCIENCE ABC, Journal of Isfahan University of Medical Sciences*).

It is assumed that journals with titles containing terms of geographical regions are mainly concerned with problems common for these regions. Good examples, in this regard, are *Iranian Journal of Medical Sciences* and *Archives of Iranian Medicine*, where correctly declared editorial policy and scope oriented toward local issues (eg, common diseases, history of medicine, medical journalism) allow publication of articles of interest to the local medical community, indexing in global prestigious databases (14,15) and increasing the rate of relevant citations.

Journal titles with the term ‘international’ imply much wider geographical representation and scope of interests. For example, *Scandinavian Journal of Rheumatology* frequently takes for publication manuscripts written by authors from Scandinavian and other countries on issues of great importance to the Scandinavian region (ie, inflammatory arthritides). Publications on diseases rare in this region but common elsewhere (Behcet disease, familial Mediterranean fever) rarely find their home in this journal. In contrast, *Rheumatology International* publishes articles on a wide range of rare and common rheumatic diseases and therefore attracts authors from all over the world.

Wide scope of interests is an advantage for a journal pursuing wider visibility. In some cases, the expansion of interests sometimes associated with changing a journal’s title and language can result in internationalization and substantial growth of the journal’s scientific prestige. The relevant examples are high-impact journals such as *Rheumatology* (formerly *British Journal of Rheumatology*), *Heart* (formerly *British Heart Journal*), and *Journal of Cardiovascular Medicine* (formerly *Italian Heart Journal*).

Original content of high scientific merit is critical for both visibility and prestige. Journals, particularly small ones, publishing duplicate articles, items on topics extensively covered by other journals, news notes, and (dubious) advertisements are disadvantaged in terms of scientific prestige and visibility in most online databases. Citability of a journal and its chances for inclusion in highly selective databases such as MedLine are reduced when editors give preference to the abstracts of meetings. The scientific merit of most biomedical journals diminishes when articles with a low level of evidence (ie, articles on inconclusive data, biased expert opinion notes, letters, and case reports) are published in substantial proportions, negatively affecting the journals’ prestige and chances for future citations. The latter is particularly threatening for newly launched and small journals with limited indexing. In contrast, most big journals such as *The Lancet and The New England Journal of Medicine* actively publish peer reviewed, well documented, unique, comprehensively discussed, and educational case reports, which, apart from being highly readable, affect marketing of new drugs, enhance pharmacovigilance (detection of adverse drug events) and timely diagnosis of rare disorders (16). Interactive communication between authors and readers and publication of letters-to-the-editors and commentaries on recently published materials exemplifies good editorial work (17-19). Unfortunately, the majority of recently launched biomedical journals, due to financial limitations and in order to provide more space for regular articles, have abandoned editorials and communication letters as indicators of comprehensive editorial work.

## Indexing and journal visibility

Indexing of a scholarly journal in databases, catalogs, and libraries relevant to the subject of the journal is critical for enhancing its quality, attracting audience, and increasing citations. Nowadays, there are many general and specialized indexing services used for ranking journals, individuals, research and academic institutions and even countries. Of these, the most popular and prestigious are indexing services offered by Thomson Scientific (formerly the Institute for Scientific Information). Most scientific journals are either striving to get access to its highly selective Science Citation Index Expanded database or to get the highest possible 2-year Journal Impact Factor (JIF) published by Journal Citation Reports. JIF is viewed as a surrogate measure of journal quality by some experts (20). However, it is largely accepted that journal quality should not be judged based on a single measure (21,22). Almost all proposed quality measures have limitations and some, including the notorious 2-year JIF and H-index, can be manipulated by editors and authors (23-26). As an alternative to JIF, the recently proposed SCImago Journal Rank (SJR) may be a more accurate measure of journal quality, which is particularly not influenced by self-citations and is calculated based on SCOPUS database (27,28).

It seems obvious that editors’ efforts should be directed toward improving publication standards and getting access to many relevant indexing services. Editors of the journals rejected by leading general online databases should put more efforts into attracting better quality publications, approaching alternative indexing services and widening visibility in open access repositories, regional, specialized, and sub-specialized databases.

Wider visibility can become a gateway for indexing by prestigious indexing services and libraries. Although there are no specific recommendations, the least editors can do to increase journal visibility and citation chances is to publish comprehensive instructions for authors and to check title pages for inclusion of accurate and informative titles, each author’s affiliation, full address for postal and electronic correspondence, abstracts, keywords, citation options using Uniform Record Locators (URL), and validated references (29). This information is required for correct indexing, facilitating retrieval of appropriate sources, and scientific ranking of individuals, institutions, and countries in the prestigious databases such as Web of Science, Scopus, and PubMed.

Perhaps the most difficult task is to correctly choose keywords. Reflective words or phrases can be found in the main text of the manuscript. Ideally these should be similar to the indexing terms listed in the relevant vocabulary thesauri such as Medical Subject Headings (MeSH) used by the US National Library of Medicine for indexing articles in PubMed (30). A number of other controlled thesauri are used for indexing articles in Scopus: EI thesaurus (covers engineering, technology and physical sciences), Emtree (life sciences and health sciences), Geobase Subject Index (geology), Regional Index (geography), Species Index (biology), etc (31).

Reference validation and correct listing is yet another sensitive issue, implicating the correctness of citation tracking and calculation of journal impact factors by Scopus and Web of Science. First of all, it is necessary to choose the format for the references. Both the Harvard and Vancouver systems are widely used, with a preference given to the latter. In the Vancouver system, references are listed in chronological order of their citation in the text and quoted by Arabic numbers (32). Unless absolutely necessary, authors should be advised not to cite manuscripts submitted for publication, unpublished and invisible on the Internet sources and to replace abstracts of meetings, dissertations and old sources with recently published articles. Accepted journal articles published in ahead-of-print format can be cited using their Digital Object Identifiers (DOI). Citations to Internet sources should give exact URLs with date of access, as some may change their location or disappear with the time (33,34). Based on the available evidence on common errors in journal article citations, editors should verify all parts of the cited sources, paying particular attention to the spelling of authors’ names, journal and article titles (35,36). To avoid any loss of citations while indexing, correct abbreviations of journal titles can be retrieved from relevant databases (eg, PubMed/MedLine). Reference validation can be performed manually or using electronic editorial management tools.

## Timeliness of publication

Timeliness of online and print publication of journal issues and its separate articles is greatly valued by most leading indexing/archiving databases. Quality and rate of submissions, availability of qualified and responsible reviewers, time spent on correspondence between editors, reviewers, and authors, use of electronic editorial management software, and publishing/funding agency may all have implications for the publication schedule (37-39). As a message for journals striving to tackle delays in publishing, evidence based on the peer review in *Croatian Medical Journal* suggested that timeliness of a small journal publication can be reached by investing more on education of local reviewers, particularly female experts (40).

Rapid publication is critical for maintaining the interest of contributors and readers in biomedical sciences. *The Lancet, The New England Journal of Medicine,* and some other high-impact journals have developed a policy for prioritizing fast-track publication of peer-reviewed manuscripts with potentially significant impact on human health (ie, those on randomized controlled trials concerning new drugs and technologies and systematic reviews of these trials) (41,42). As a result, these journals have boosted their scientific prestige, 2-year JIF, and immediacy index, reflecting how rapidly publications from a journal are cited within the same year of publication (13).

## Conclusion

To ensure the quality of scholarly publications, editors along with authors, reviewers, and the publisher should pay close attention to every detail, starting from the submission to publishing the final scientific product. Understanding of the role of biomedical journals in disseminating cutting edge information, educating readers, stimulating research activity, and influencing medical practice obliges all those taking part in science editing and publishing to assess available resources and to direct efforts at expanding international outreach and further promoting communication between experts. The process of improving quality of the journal is continuous and equally important for top and low rank, big and small, and general and specialized journals. While well established and widely visible journals are aiming to maintain high editing and publishing standards, new and small journals are struggling to meet basic requirements and to break the vicious cycle of inadequacy associated with poor scientific quality and infrequent submissions in substandard English, lack of international collaboration, inexperienced reviewers, poor representation in relevant libraries and online catalogs, etc (43). Of course most confounding factors of inadequate quality of publications are grounded on insufficient funding. However, there are many other genuine causes preventing good research data from representation in PubMed/MedLine and Web of Science. Of these, the lack of expertise, interest and responsibility of editors of unsuccessful journals is of prime concern (44). As a result of inadequate editorial policies and management, journal articles contain outdated, not properly structured and synthesized information, are based on numerous statistical and ethical flaws, hardly attract readership, and prevent the journal from indexing in global prestigious databases (44).

To overcome poor biomedical science editing, several possible solutions can be suggested. Educating editors and reviewers seems to be the most feasible task which can be fulfilled by professional associations such as WAME, COPE, CSE, EASE, regularly issuing guidelines, publishing teaching materials, organizing online discussions for its members, and arranging scientific and educational meetings worldwide. Regional medical editors associations such as those in the Eastern Mediterranean, Asian Pacific, and some other regions can also play important supportive role. A new approach to improving the quality is emerging in a form of evidence-based biomedical journalism which may distinguish “good” and “bad” journals by employing the established principles of evidence-based medicine (45).
